# Socio-economic patterning of food and drink advertising at public transport stops in Edinburgh, UK

**DOI:** 10.1017/S1368980021004766

**Published:** 2022-05

**Authors:** Tony Robertson, Ruth Jepson, Kyle Lambe, Jonathan R Olsen, Lukar E Thornton

**Affiliations:** 1 Biological and Environmental Sciences, Cottrell Building, University of Stirling, Stirling FK9 4LA, UK; 2 Scottish Collaboration for Public Health Research and Policy, University of Edinburgh, Edinburgh, UK; 3 MRC/CSO Social and Public Health Sciences Unit, University of Glasgow, Glasgow, UK; 4 School of Exercise and Nutrition Sciences, Deakin University, Geelong, Australia; 5 Department of Marketing, Faculty of Business and Economics, University of Antwerp, Antwerp, Belgium

**Keywords:** Advertising, Marketing, Unhealthy commodities, Inequalities, Deprivation, Spatial

## Abstract

**Objective:**

Outdoor advertisements for food and drink products form a large part of the food environment and they disproportionately promote unhealthy products. However, less is known about the social patterning of such advertisements. The main aim of this study was to explore the socio-economic patterning of food and drink advertising at bus stops in Scotland’s capital city, Edinburgh.

**Design:**

Bus stop advertisements were audited to identify food/drink adverts and classify them by food/drink category (i.e. ‘advert category’). This data were then linked to area-based deprivation and proximity measures. Neighbourhood deprivation was measured using the bus stop *x*/*y* co-ordinates, which were converted to postcodes to identify the matching 2012 deprivation level via the Scottish Index of Multiple Deprivation. Distance to schools and leisure centres were also collected using location data. Generalised estimating equations and linear regression analyses were used to assess associations between the promotion of advert categories and deprivation and proximity to schools/leisure centres, respectively.

**Setting:**

Edinburgh city, United Kingdom.

**Results:**

561 food/drink advertisements were identified across 349 bus stops, with 8 advertisement categories noted and included in the final analysis, including alcohol, fast food outlets and confectionary. The majority of adverts were for ‘unhealthy’ food and drink categories, however there was no evidence for any socio-economic patterning of these advertisements. There was no evidence of a relationship between advertisements and proximity to schools and leisure centres.

**Conclusions:**

While there is no evidence for food and drink advertising being patterned by neighbourhood deprivation, the scale of unhealthy advertising is an area for policy evaluations and interventions on the control of such outdoor advertising.

There is a wide range of factors influencing diet and food choice. A key area of interest over recent decades has been the influence of the ‘food environment’, including the availability, affordability and marketing of food and drinks and how food environments may be associated with neighbourhood deprivation^(1,2)^. It is typically hypothesised that unhealthy food and drink are more available, affordable and more frequently marketed in more deprived areas. There is mixed evidence across the literature that food stores such as supermarkets are more prevalent in affluent areas^(3–5)^, but there does seem to be consistent evidence for the higher prevalence of fast food and takeaway outlets in deprived neighbourhoods^(6)^. One Australian study has found that more unhealthy products were advertised in deprived neighbourhoods^(7)^, although this has not been replicated in the UK^(8,9)^.

In the UK, advertising of tobacco products has been banned since 2003^(10)^, but other products are largely unrestricted. There are a few exceptions such as a UK code of practice for the advertisement of high in fat, salt or sugar products that appeal to children^(11)^. It has also been reported that the top 18 UK brands associated with crisps, confectionery and sugary drinks spent more than £143 m on advertising their products in 2016, leading to third sector (charities, social enterprises and voluntary groups) and health organisations to call for the restriction of advertising of unhealthy food products^(12)^. Promotional material may be targeted towards people of a particular gender, age group, nationality or even people with specific education levels, interests or behaviours^(13)^. Advertisements can also be targeted towards people living in a specific area, with outdoor (out-of-home) advertising representing a potentially effective method, both in terms of cost and impact^(14–16)^. It has been shown that food preferences and purchasing and consumption habits may be influenced by such targeted advertising^(17–19)^.

As viewing outdoor advertising is non-discretionary, the placement of posters or billboards can effectively advertise to large groups such as the drivers who pass by them, creating a large exposure group, but also to those who live, study or work in close proximity to their location and will see them regularly^(20–22)^. Outdoor advertisements for food/drink products form a large part of the food environment, with outdoor advertising disproportionately promoting unhealthy (*v*. healthy) and nutritionally poor foods, especially around schools and areas that children frequent^(23–25)^.

Advertisements placed at outdoor public transport stops are one type of advertising through which socio-economic targeting by area may be possible. For example, those experiencing higher levels of deprivation may be more reliant on public transport such as the bus network and this more exposed to this form of outdoor advertising^(26)^. A small literature exists, mainly from Australia, that has focused on transit stops as key means of outdoor advertising of unhealthy products, including food^(7,27)^. However, there remains limited evidence for the socio-economic patterning of outdoor food advertising via transit systems in other contexts.

The aim of this study was to explore the socio-economic patterning of food advertising at transit stops, focusing on bus stops (a highly utilised form of public transport in this context) throughout Scotland’s capital city, Edinburgh. Our hypothesis was that there would be a higher prevalence of unhealthy food advertising (fast food, soft drinks, confectionary etc.) and lower prevalence of healthy food advertising (water, fresh fruit and vegetables, etc.) situated in more deprived areas within the city compared with areas with lower levels of deprivation. However, we also considered the relationship between advertising and distance to leisure centres and schools as a possible alternative explanation for the patterning of unhealthy food/drink advertisements.

## Methods

### Advertising locations

This study took place in the City of Edinburgh, Scotland across all 17 electoral wards in the city (spatial units used in the UK to elect local government in metropolitan and non-metropolitan districts). Edinburgh is Scotland’s second largest city (2018 population = 518 500)^(28)^ and while it has lower than average deprivation compared with Scotland as a whole, there remain areas of considerable deprivation^(29)^. Given the limited tram and train services provided in the city, this study focused on bus transport. Data provided by Edinburgh City Council allowed identification of 2227 bus stops, 447 of which had advertising (20 %). JCDecaux, the private company responsible for advertising, provided *x*/*y* coordinates for the exact location of each bus stop, along with an ID number, street name, post code and closest house number or point of interest to the stop (e.g. ‘before’ or ‘after’ a junction with another street, or a building such as a shopping centre or tourist centre). Details of specific advertisements were not available without visiting the stops.

### Advertisement audit (using ODK collect)

‘ODK collect’ (OpenDataKit) is an open-source smart device application that allows individuals to create tailor-made survey forms which can then be filled in and saved to the device’s internal memory while in the field, and are uploaded to the server when connected to a Wi-Fi network. This enabled us to create a list of questions to be answered about each advertisement while present at that location, including confirming the *x*/*y* coordinates of the bus stop, the type of area it was located in (residential, industrial etc.), details about the food or drink product being advertised and whether a price or special offer was featured in the advertisement. The form used in this research adapted the auditing tool created by Settle *et al*.^(7)^ From this tool, 10 main food categories were created with a small number of edits to reflect differences in food advertising and availability in the UK as opposed to Australia. For example, a ‘flavoured water’ option was added to ‘Cold Beverages’, while ‘Pies/Savoury Pastries’ was added to ‘Energy-Dense Snacks’. These 10 categories were: cold beverages, hot beverages, energy-dense snacks, fast foods (brand and product), breakfast cereals, dairy, other hot food (e.g. soup), fruit and vegetables, other food products not previously identified (free text) and food stores. The full list of individual products audited is available in online Supplemental Table 1. Given low sample sizes in some categories, we re-categorised the 10 original categories into 8 for the final analyses, as was a simpler 4-category construct. The 8 categories were alcohol, sugary drinks (soft drinks, diet soft drinks, energy drinks, sports drinks, flavoured milk, flavoured water and iced tea), fruit juices (fruit smoothies, concentrate juice drinks and fruit cordials), iced coffee, confectionary, frozen desserts, fast food outlets and food stores. For the 4-category version alcohol, sugary drinks, fruit juices and iced coffee were combined into cold beverages. Confectionary and frozen desserts became energy-dense snacks while fast food outlets and food stores remained unchanged.

### Data collection

Data collection was carried out between 17th June and 11th July 2015 by one researcher (KL) travelling to each relevant bus stop. A tablet computer was used to record data about each advertisement present on bus stop posters or digital adverts. Mobile internet was used to obtain and store more accurate GPS data on the location of each stop and also to access the bus stop map to ensure that the planned route was adhered to, or to deviate from the route when suitable to collect more data on a given day.

### Statistical analysis

*X*/*y* coordinates were converted to postcodes using the address of the closest building to the plotted point. The postcode associated with each stop was used to determine the neighbourhood socio-economic deprivation using the 2012 Scottish Index of Multiple Deprivation (SIMD 2012). Scottish Index of Multiple Deprivation scores were converted into quintiles, with group 1 comprising the most deprived 20 % and group 5 the 20 % least deprived neighbourhoods in Scotland. Using the bus stop postcodes also allowed for the calculation of population density of each electoral ward based on population size and ward size (km^2^) (see online Supplemental Table 2), supplied by the Scottish Local Government Boundary Commission.

**Fig. 1:**
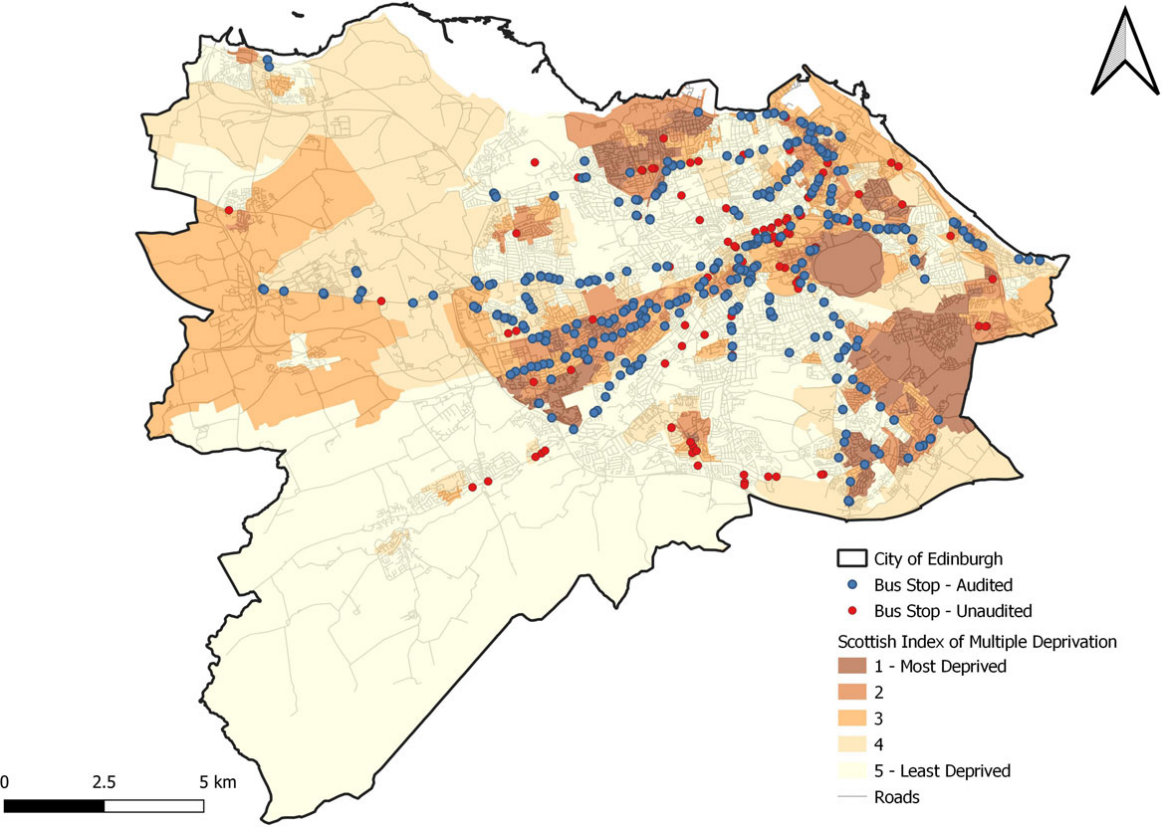
Percentage of all sampled adverts from each food category and for each deprivation quintile. Note: percentages for the whole figure add up to 100 %

Statistical analysis was performed using SPSS version 21 (IBM Corp.). Descriptive statistics were calculated to determine the mean number of food adverts per bus stop and describe the frequency of food and drink advertisements by advert category (Fig. 1). χ^2^ analysis was used to test for deprivation differences in stops sampled *v*. not sampled. Then generalised estimating equation models were used to compare the patterning of food advertisements by area-level deprivation (Scottish Index of Multiple Deprivation) allowing for repeat measurements as multiple advertisements were often located at the same bus stop. For each food category, a separate model was run using a binary dummy dependent variable (e.g. alcohol adverts coded 1, other adverts coded 0) and deprivation (Scottish Index of Multiple Deprivation quintiles) as the independent variable. Models were run with and without adjustment for population density as a proxy for potential footfall. Results reported are OR, where an OR above 1 indicates higher odds of that advert appearing in a less deprived area.

We also considered the relationship between advertising and distance to leisure centres (public sport and exercise facilities) and schools as a possible alternative explanation for our findings from the socio-economic patterning analysis. Previous research has identified that sugary drinks and alcohol are heavily advertised around schools and other areas children are likely to frequent^(30)^. All Scottish school locations were downloaded and geocoded using their full UK postal code within ArcMap 10·3^(31)^. Leisure centre locations were downloaded from Ordnance Survey as polygon shape files and subsequently converted to point locations for proximity analysis using the ‘convert features to points’ tool within ArcMap^(32)^.

Network distance analysis was performed for each bus stop location to their nearest school and leisure centre on the integrated transport network^(33)^. The network analysis measured the distance of each bus stop to all schools and leisure centres within Edinburgh and the final output provided the nearest school and leisure facility to all bus stops and the distance (km) between the 2 locations. The analysis was performed using the closest facility tool within Network Analyst (ArcMap 10·3, ESRI).

The median distance (km) and IQR for bus stops to their nearest school and leisure centre are described. Linear regression was performed for each bus stop distance as the independent variable (distance to schools or leisure) by the number of advertisements in each individual category (dependent variable). The analysis was performed for all bus stops and only bus stops that had a facility within a 0·5 km network distance. We applied a network buffer of 0·5 km as this is a commonly applied distance that is deemed walkable as a transport choice^(34)^.

## Results

**Fig. 2:**
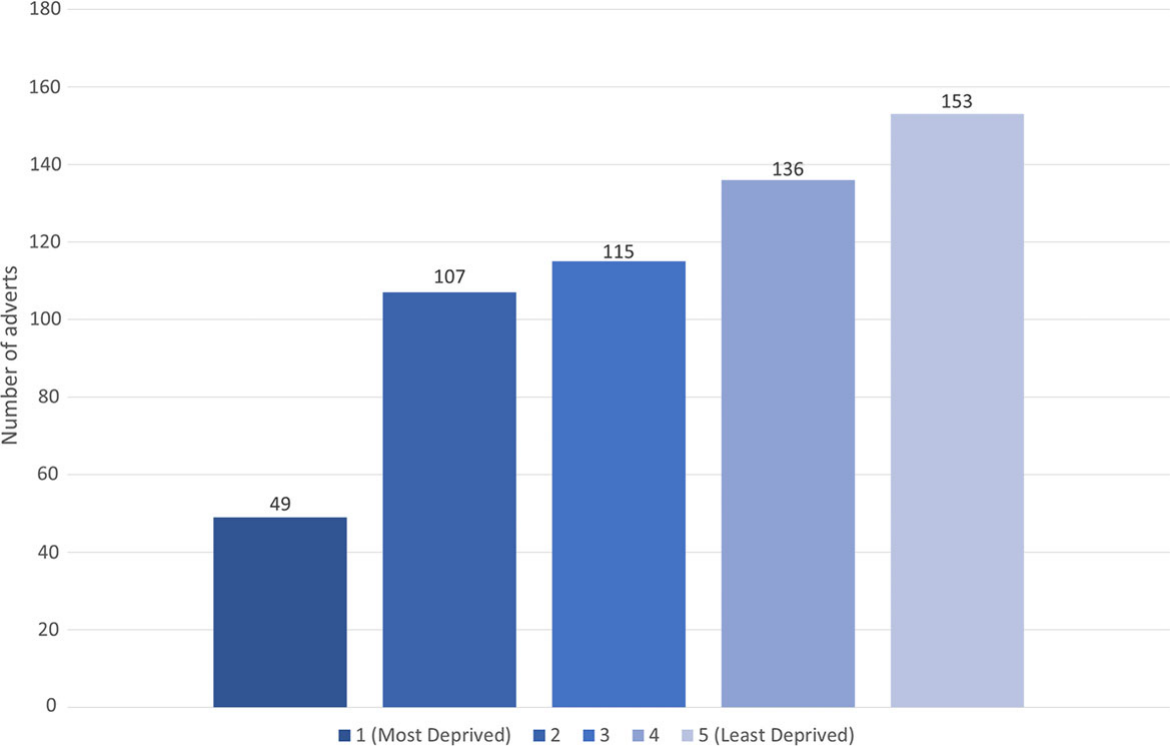
Map of bus stops with advertisements across Edinburgh city. Red pins represent bus stops visited during data collection. Blue stars denote stops not sampled

**Fig. 3:**
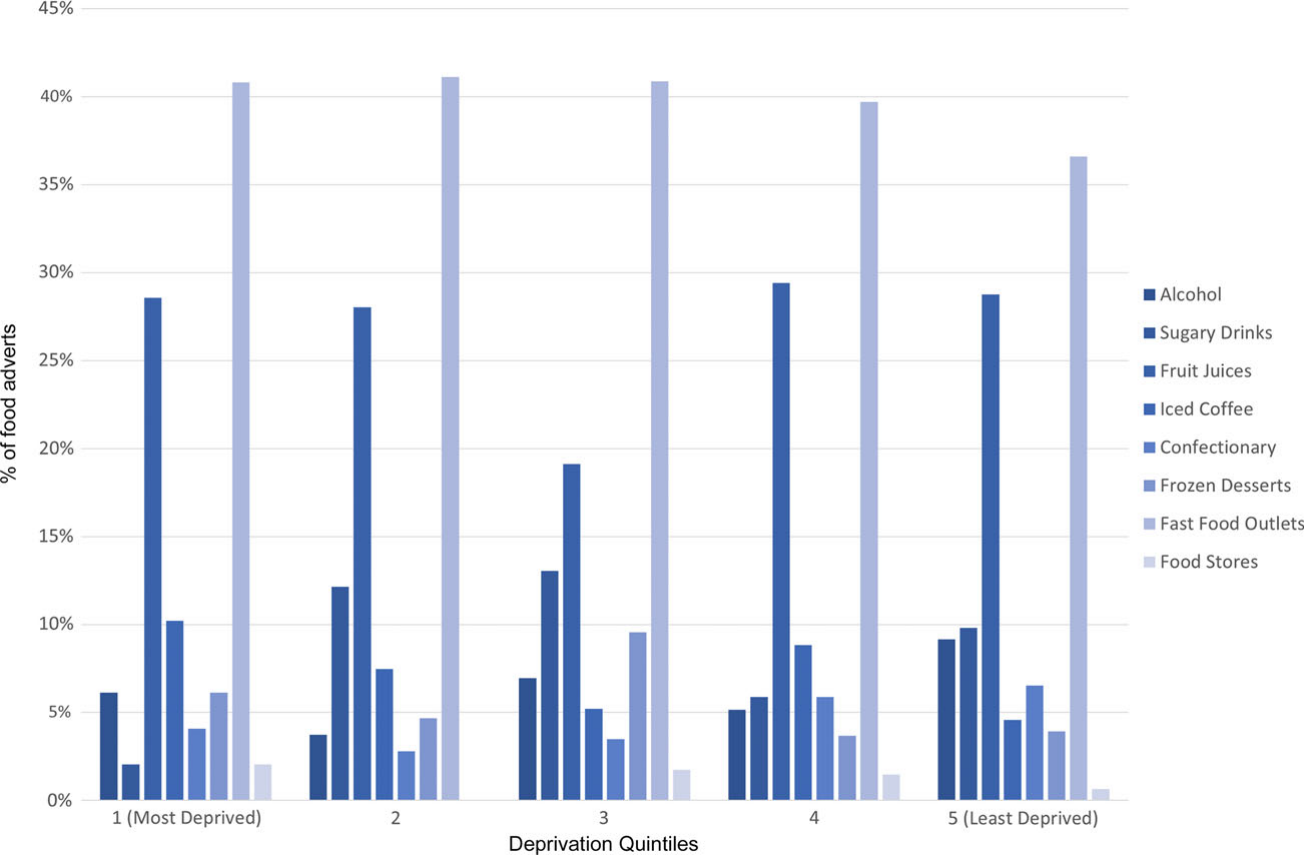
Number of food adverts by deprivation quintile

**Fig. 4:**
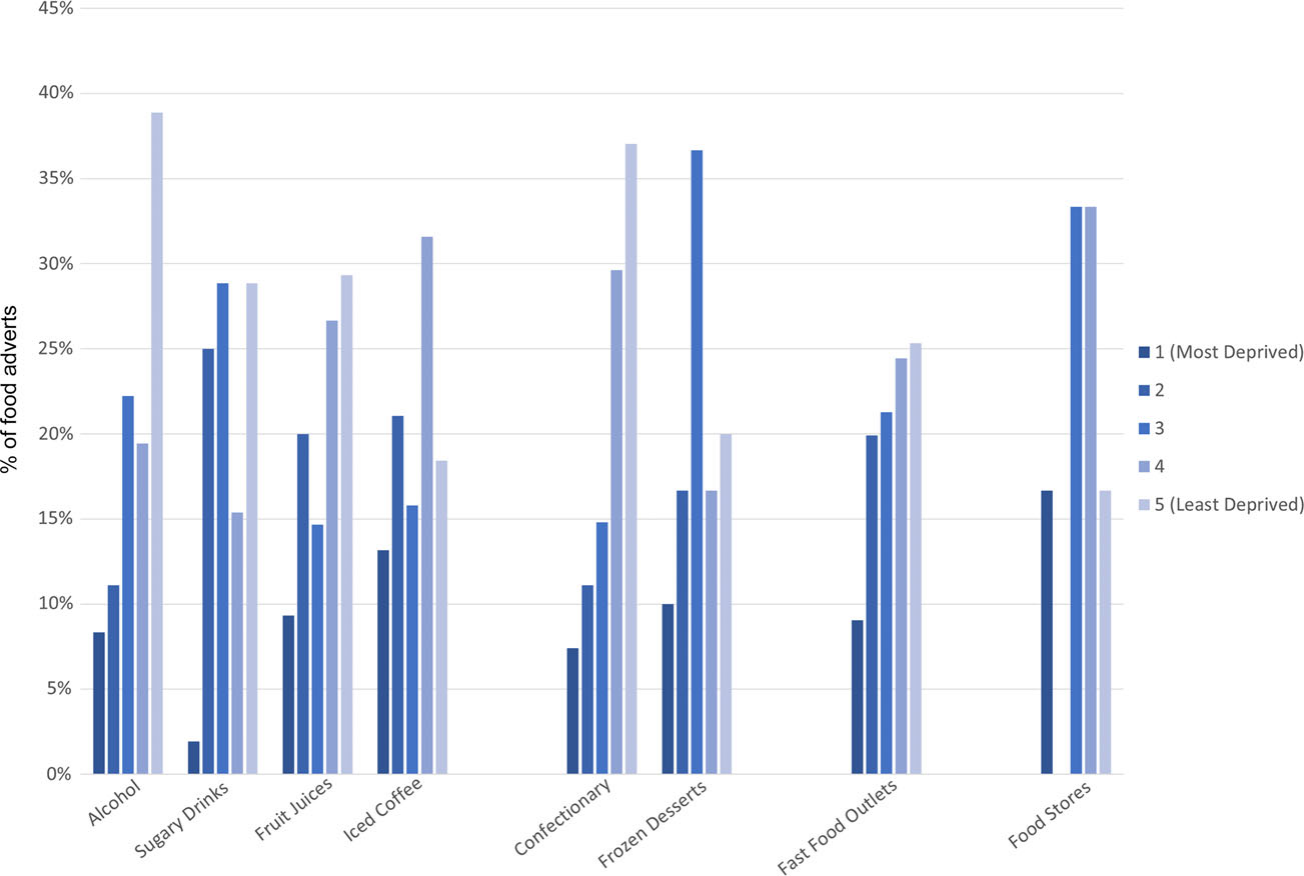
Percentage of adverts present within each deprivation quintile, broken down by food category. Note: percentages for each food category add up to 100 %

Of the 447 bus stops in the city with advertising infrastructure present, data were collected from 349 stops, with 59 bus stops not available due to refurbishments and the remaining 39 bus stops not visited due to field work time constraints or access issues (e.g. closed roads) (Fig. 2). After data cleaning, complete data were only present for 343 of the 349 bus stops, although some bus stops had multiple advertisements. This equates to 77 % (343 of 447) of available stops being sampled from and included in the analysis. There was no difference in terms of deprivation profile for sampled *v*. not sampled stops (*χ* ^2^, *P* = 0·096). Of the 343 bus stops visited, only 298 had advertisements on them at the time. In total, 561 food advertisements were recorded across these 298 bus stops (mean = 1·88 food advertisements/stop). Fruit juices and fast food outlets made up 66 % of all adverts (Table 1). There were more food and drink adverts in the least deprived areas and a social gradient across all 5 quintiles (Fig. 3). As a percentage of adverts within each quintile though, descriptive analysis suggested no clear social patterning in any of the 8 food categories (Fig. 4).

**Table 1:** Generalised estimating equation models showing likelihood of bus stop food/drink advertising according to deprivation level

	OR*	LL 95 % CI	UL 95 % CI	*P*
Cold beverages (*n* 276)	0·851	0·705	1·027	0·093
Alcohol (*n* 36)	0·827	0·627	1·090	0·177
Sugary drinks (*n* 52)	0·986	0·806	1·208	0·893
Fruit juices (*n* 150)	0·988	0·733	1·332	0·936
Iced coffee (*n* 38)	1·152	0·918	1·446	0·223
Energy-dense snacks (*n* 57)	0·971	0·794	1·186	0·770
Confectionary (*n* 27)	0·796	0·575	1·102	0·170
Frozen desserts (*n* 30)	1·151	0·901	1·471	0·261
Fast food outlets (*n* 221)	1·054	0·983	1·131	0·139
Food stores (*n* 6)	1·059	0·592	1·896	0·847

* An OR above 1 indicates higher odds of that advertising category appearing in a less-deprived areas.

The generalised estimating equation analysis found that for each category there were no associations between deprivation and food advertising categories. For example, there was no difference in the odds of a cold beverage advertisement (*v*. all other advert categories) in more deprived areas (OR: 0·85, (95 % CI 0·71, 1·03), *P* = 0·093) (Table 1).

For the proximity analysis, the median distance of bus stops to the nearest school and leisure centre was 0·63 km and 1·30 km, respectively (Table 2). For bus stops that were less than 0·5 km from a school, for each unit increase in proximity to that school (i.e. the closer one gets to a school), there were higher numbers of energy-dense snack advertisements, although the effect size is small (Coef: 0·058, (95 % CI 0·01, 0·11), *P* = 0·035). When bus stops located further than 0·5 km from a school were considered, this association was no longer present. No differences in advertisements were seen when examining bus stops less than 0·5 km from a leisure centre. The same was true when examining bus stops located further than 0·5 km from a leisure centre (for full results, see Table 2).

**Table 2:** Regression models for the likelihood of food/drink advertisements being present close to schools or leisure centres

	Median		IQR	
a) Bus stop distance from nearest school and leisure centre (km)				
School	0·63		0·03 to 3·28	
Leisure centres	1·30		0·002 to 3·54	
b) Schools				
i) Any distance	Coefficient	Lower limit 95 % CI	Upper limit 95 % CI	*P*
Cold beverage	−0·269	−1·248	0·710	0·589
Energy-dense snack	0·004	−0·138	0·146	0·955
Fast food	−0·045	−0·133	0·043	0·315
Food store	0·222	−0·181	0·625	0·279
ii) Less than 0·5 km				
Cold beverage	0·160	−0·067	0·388	0·166
Energy-dense snack	0·058	0·004	0·112	0·035
Fast food	0·008	−0·026	0·043	0·629
Food store	0·032	−0·130	0·194	0·699
c) Leisure centres				
i) Any distance				
Cold beverage	0·374	−1·286	2·033	0·658
Energy-dense snack	−0·140	−0·381	0·101	0·253
Fast food	−0·065	−0·215	0·084	0·393
Food store	−0·155	−0·839	0·528	0·656
ii) Less than 0·5 km				
Cold beverage	n/a			
Energy-dense snack	0·014	−0·102	0·129	0·815
Fast food	0·028	−0·056	0·112	0·505
Food store	0·185	−0·117	0·487	0·224

## Discussion

This study identified that food and drink advertisements, the majority of which were unhealthy and high in fat, salt and sugar categories, were prevalent across the study site of Edinburgh’s bus network, however, there was no evidence for any socio-economic patterning of unhealthy advertisements. There was also no evidence of a relationship between food advertisements and proximity to areas where children are likely to be (schools and leisure centres).

To date, there has been a small number of papers focused on inequalities in transport advertising of food/drink products, predominantly situated in Australia. Settle *et al*. assessed food advertisements at public transit (bus, tram and train) stops within suburbs in Melbourne^(7)^. Although there was a similar amount of food advertisements present at transit stops in the least and most deprived suburbs, advertisements for fast food, flavoured milk and fruit juice were more common in the most deprived areas. Sainsbury *et al.* ^(35)^ audited all 178 train stations on the Sydney metropolitan train network. They found the majority of food/drink adverts were in unhealthy categories (snack foods and sugar-sweetened beverages). The proportion of advertisements that were for unhealthy foods (as a function of all advertisements) was highest in more deprived areas.

The study presented here does not mirror the evidence from Australia for a socio-economic patterning of unhealthy food and drink products on transit stops. Both Australian studies sampled either larger areas and/or for longer time periods. For example, Sainsbury *et al.* ^(35)^ sampled in both summer and winter, whereas this study only occurred during the UK summer. The Australian studies also focused on train networks rather than buses and it is possible that bus and train networks differ in their service to mixed socio-economic areas and how they are selected by advertisers. The origins of how cities are constructed, how areas of deprivation emerge and are maintained and how transport networks link to such deprivation may also be very different between these contexts. In terms of UK studies, Adams *et al*.^(8)^ sampled all outdoors advertisements in Newcastle, finding that total advertising and food advertising space was largest in the most deprived areas. However, there was no evidence of socio-economic patterns in the category of foods being advertised.

Recently Olsen *et al*.^(9)^ have published evidence from bus stop adverts across the central belt of Scotland (including the cities of Edinburgh and Glasgow) using Google Maps to facilitate the advertising audit. That study also found that no social patterning of food/drink advertisements and food/drink advertisements were no more likely to be located near schools. However, using individual mobility data from school-aged children, they found that children who lived in more deprived areas had greater contact with the transport network (e.g. via walking along major bus routes) and were more likely to be exposed to unhealthy food/drink product advertising during their commutes to school and leisure time compared with those from more affluent areas.

It is possible that advertisers are not specifically advertising unhealthy products in more deprived areas in Edinburgh and other UK cities using bus stops. However, this does not exclude the possibility that unhealthy advertisements across the outdoor environment are being disproportionately targeted at lower socio-economic groups. It may be that the combination of the whole environment people come into contact with on a regular basis is more important, going beyond bus stops to include advertisements on other transit-type stops, vehicles and tickets, as well as billboards, on people’s phones, televisions and simply encountering these shops/products on the street. It remains a challenge to accurately and efficiently measure the advertising that people see from all ‘angles’, at work/school, at home, during leisure time and during their commutes. There is evidence that more deprived neighbourhoods are more obesogenic than their more affluent counterparts and that those from more deprived areas commute more (at least pre-COVID) by bus^(26)^, however it remains unclear if this extends to the advertising sphere of the built environment^(36)^. Anecdotal evidence collected during fieldwork from Lothian Buses staff suggested that bus stops with shelters (that have space for advertisements) are less common in more deprived areas due to a perceived risk of vandalism compared with simpler bus stops which contain a pole, timetable and no seating/stop area for waiting passengers. While this anecdotal evidence has not been verified with data in our study, it does match with our findings that there are increasing numbers of adverts with increasing affluence. It may be that in the UK context, and with ‘exposed’ advertising structures, that there could be a reverse social patterning taking place driven by the presence or absence of advertising.

Some other considerations to aid interpretation of the results include the possibility that Edinburgh is not the best-suited city for assessing inequalities, as it generally has lower levels of deprivation compared with many other areas, particularly the other cities of Glasgow and Dundee^(29)^. Further, perhaps other cities with a wider range of transport systems would provide different results. Finally, the description of products as ‘unhealthy’ is limited and can be confusing. For example, fruit juices could be perceived as both healthy (a source of nutrients) and unhealthy (high sugar content). In our context, most of the fruit juice advertisements recorded were for sweetened fruit smoothies and concentrated juice drinks, both of which are high in sugar. Therefore, we deemed these to represent ‘unhealthy’ food advertisements.

This study was built off the strength of novel data collected ‘on-the-ground’, allowing the identification of multiple advertisements at the same bus stop (e.g. rolling, poster-based advertisements or digital advertisements). The data collection was strengthened and made more efficient by the inclusion of *x*–*y* coordinates of each bus stop with advertisements (provided by the company responsible for managing all advertisements on bus stops in the city). Analysis was strengthened by combining the data collected with routine data such as deprivation indices, population sizes and proximity to schools and leisure centres.

The key weakness of this study was that it was limited to one city at a single time-point and only focused on one type of outdoor advertising. The adverts displayed do also change over time so only offer a snapshot of the advertising present. The data were collected in one summer period, so we cannot rule out seasonal variations. However, given the limitations in human and financial resources, this pragmatic data collection method did still allow for the collection of over 350 different advertisements in a relatively short time-period. Future studies could be improved by: providing a wider focus than just the city of Edinburgh; the inclusion of repeat measures across a calendar year and broadening the focus to include other forms of outdoor advertising (e.g. billboards).

Despite the lack of evidence for a social patterning of unhealthy food advertisements, or other potential associations with proximity to schools and leisure centres, it was evident that the vast majority of advertisements promoted unhealthy food categories. Recent policy changes in London have seen a ban on ‘junk food’ advertisements on all London Transport Network facilities and vehicles (trains, the underground and buses)^(37)^. In Scotland, following a consultation, the Scottish Government set out in its Programme for Government (2019–2020) that it would bring forward a Bill on Restricting Food Promotions within the year’s legislative programme^(38)^. How effective these policies are at reducing exposure to high in fat, salt and sugar foods, especially in children, is not yet known but is an example of future research that can help us understand the effects of these advertisements (or the lack of them) on health behaviours and how advertisers target specific areas/people.

## Supplementary material

For supplementary material accompanying this paper visit https://doi.org/10.1017/S1368980021004766. click here to view supplementary material
