# Supplementary material for: Socio-economic patterning of food and drink advertising at public transport stops in Edinburgh, UK
Source: Public Health Nutr. 2021 Dec 10;25(5):1131–9. doi: 10.1017/S1368980021004766 (PMC7612707; doi:10.1017/S1368980021004766)
Supplement: Supplementary file 1 [file S1368980021004766sup001.docx]

**SUPPLEMENTARY FIGURES AND TABLES**

**Supplementary Table 1**  Food and drink categories used in ODK collect tool

| **Cold Beverage** | **Hot Beverage** | **Energy Dense Snack** | **Fast Food (brand and product)** | **Breakfast Cereals** | **Dairy** | **Other hot food** | **Fruit & Vegetables** | **Other Food Product (brand and product) – free text** | **Food Store (store name) – free text** |
| --- | --- | --- | --- | --- | --- | --- | --- | --- | --- |
| Soft drink e.g. Coca Cola | Tea | Crisps | Fast food outlet | ‘Healthy’ product (high fibre/low sugar) | Cheese | Noodles | Fruit |  |  |
| Diet soft drink  e.g. Diet Coke | Coffee | ‘Low fat/healthy’ crisps | Fast food product | ‘Unhealthy’ product (low fibre/ high sugar) | Yoghurt | Soup | Vegetables |  |  |
| Energy drink  e.g. Monster | Hot chocolate | Chocolate |  |  | Full-fat milk (not flavoured) | Chips/ wedges etc. |  |  |  |
| Sports drink  e.g. Lucozade |  | Confectionary |  |  | Low/no-fat milk (not flavoured) |  |  |  |  |
| Flavoured milk e.g. Frijj milkshake |  | Ice cream/frozen desserts |  |  |  |  |  |  |  |
| Fruit juice e.g. fruit smoothies, concentrate juice drinks and fruit cordials |  | Biscuits |  |  |  |  |  |  |  |
| Iced tea |  | Cereal bars |  |  |  |  |  |  |  |
| Water |  | Doughnuts |  |  |  |  |  |  |  |
| Flavoured water e.g. Volvic Touch of Fruit |  | Cakes |  |  |  |  |  |  |  |
| Alcohol |  | Mints/gum |  |  |  |  |  |  |  |
|  |  | Sugar free mints/gum |  |  |  |  |  |  |  |
|  |  | Pies/savoury pastries |  |  |  |  |  |  |  |

**Supplementary Table 2** Ward size and population size

| **Ward** | **Size (km^2^)** | **Population Size** | **Population Density (people/km^2^)** |  |
| --- | --- | --- | --- | --- |
| Almond | 62.85 | 26,571 | 423 |  |
| Pentland Hills | 79.34 | 23,842 | 301 |  |
| Drum Brae / Gyle | 7.49 | 23,643 | 3,157 |  |
| Forth | 6.82 | 33,470 | 4,908 |  |
| Inverleith | 8.07 | 32,739 | 4,057 |  |
| Corstorphine / Murrayfield | 6.63 | 22,572 | 3,405 |  |
| Sighthill / Gorgie | 7.35 | 37,478 | 5,099 |  |
| Colinton / Fairmilehead | 20.14 | 26,654 | 1,323 |  |
| Fountainbridge / Craiglockhart | 4.84 | 23,833 | 4,924 |  |
| Meadows / Morningside | 6.4 | 35,147 | 5,492 |  |
| City Centre | 4.29 | 22,838 | 5,324 |  |
| Leith Walk | 2.76 | 32,533 | 11,787 |  |
| Leith | 5.32 | 26,811 | 5,040 |  |
| Craigentinny / Duddingston | 8.52 | 25,471 | 2,990 |  |
| Southside / Newington | 6.44 | 34,801 | 5,404 |  |
| Liberton / Gilmerton | 15.74 | 33,362 | 2,120 |  |
| Portobello / Craigmillar | 10.33 | 25,735 | 2,491 |  |

**Supplementary Table 3: Number of advertisements by distance to schools and leisure centres.**

|  | **Schools** | | **Leisure Centres** | |
| --- | --- | --- | --- | --- |
|  | **Any distance** | **Less than 0.5km** | **Any distance** | **Less than 0.5km** |
| Cold Beverage | 276 | 92 | 276 | 24 |
| Energy Dense Snack | 57 | 22 | 57 | 9 |
| Fast Food | 221 | 80 | 221 | 26 |
| Food Store | 6 | 2 | 6 | 1 |
